# Increase of ALCAM and VCAM-1 in the plasma predicts the Alzheimer’s disease

**DOI:** 10.3389/fimmu.2022.1097409

**Published:** 2023-01-04

**Authors:** Jian Chen, An-Xiang Dai, Hai-Liang Tang, Chang-Hao Lu, Hao-Xin Liu, Ting Hou, Zhi-Jie Lu, Nan Kong, Xin-Yuan Peng, Kai-Xun Lin, Zi-Dong Zheng, Sheng-Liang Xu, Xiao-Fang Ying, Xiao-Yu Ji, Hui Pan, Jie Wu, Xin Zeng, Nai-Li Wei

**Affiliations:** ^1^ Department of Neurosurgery, The First Affiliated Hospital of Shantou University Medical College, Shantou, Guangdong, China; ^2^ State Key Laboratory for Medical Neurobiology, Department of Neurosurgery, Institutes of Brain Science, Fudan University Huashan Hospital, Shanghai Medical College-Fudan University, Shanghai, China; ^3^ Department of Geriatrics, The First Affiliated Hospital of Shantou University Medical College, Shantou, Guangdong, China; ^4^ Brain Function and Disease Laboratory, Shantou University Medical College, Shantou, Guangdong, China; ^5^ The Outpatient Department, Shantou Longhu People's Hospital, Shantou, Guangdong, China

**Keywords:** Alzheimer’s dementia, nomogram, ALCAM (CD166), VCAM- 1, cell adhesion molecules

## Abstract

Cell adhesion molecules (CAM) are crucial in several pathological inflammation processes in Alzheimer’s disease (AD). However, their potential for clinical diagnostics remains unknown. The present investigation evaluated the clinical significance of ALCAM, VCAM-1, NCAM, and ICAM-1 levels in the plasma of participants with cognitive impairment (44 patients with mild cognitive impairment, 71 patients with Alzheimer’s dementia, and 18 patients with other dementia) and 28 controls with normal cognitive ability. We also detected plasma levels of multiple inflammatory factors (IFN-gamma, IL-18, IL-1beta, IL-13, IL-8, IL-7, CCL11, MCP-1, TSLP, IL-10, BDNF, IL-17, IL-5, TREM-1) using Multiplex liquid chip and plasma levels of Abeta1-42 and Abeta1-40 using liquid-phase flow cytometry (FCM). Our findings demonstrated a correlation of ALCAM and VCAM-1 with age, the severity of cognitive decline, and MTA, but no significant difference between groups for NCAM and ICAM-1. ALCAM and VCAM-1 both demonstrated a positive correlation with the degree of atrophy in the medial temporal lobe structure. Further analysis revealed no significant correlation in plasma between VCAM-1, ALCAM and Abeta1-40, Abeta1-42. Nevertheless, there was a significant correlation between VCAM-1, ALCAM and many inflammatory factors. Furthermore, the predictive value of ALCAM and VCAM-1 for AD was assessed using a multi-parameter regression model. ALCAM and VCAM-1 in combination with ApoE4, education, age, and MMSE could predict AD with high precision (AUC=0.891; AIC=146.9) without imaging diagnosis. ALCAM and VCAM-1 combination improved the predictive accuracy significantly. In a nutshell, these findings revealed ALCAM and VCAM-1 as reliable indicators of Alzheimer’s disease.

## Introduction

Alzheimer’s disease (AD) is the most common dementia worldwide, with an age-standardized prevalence rate of roughly 5.7% for individuals over 60 years old. With the subtle progression of AD, patients already find themselves in the middle or late stages by the time they seek therapy, and it is difficult to delay AD deterioration. As a result, early detection, diagnosis, and treatment are critical to delaying the onset or progression of AD and lowering its incidence. Screening patients with Mild Cognitive Impairment (MCI) in the population with chronic cognitive decline can considerably benefit the early detection and prevention of AD (1). Current medical research is thus focused on developing low-cost, non-invasive, and readily accessible methods for early detection. Much effort must be expended in developing clinically feasible detection indicators, popularizing them more successfully, and making them valuable supplementary diagnostic markers (2, 3).

The three primary procedures for diagnosing Alzheimer’s disease are neuropsychological testing, imaging examinations, and biomarker detection (3, 4). Biomarkers can also shed light on specific biological changes in AD. Several biomarker detection methods have been developed today. Amyloid protein Abeta1-42/1-40 (5) and Tau-217 in cerebrospinal fluid (2, 6) have been identified as AD biomarkers. Nevertheless, it is difficult to popularize these detection methods in clinical applications due to their invasion, and they may not be utilized as a standard screening test. With advancements in detection technology, Abeta1-42/1-40, p-Tau218, p-Tau181, and Neurofilament Light Chain (NfL) may now be detected in peripheral blood (7, 8). However, all of these markers must exceed a pg/ml level. Clinical detection approaches, on the whole, fall far short of this degree of precision. In addition, they are costly and time-consuming. It is important to highlight that, while neuroimages can be incredibly useful for detecting dementia, they also have some limitations, particularly if the patient is cooperative and there are no metal compounds in the body. PET-CT is also limited in its application due to its high cost and radioactivity. Therefore, finding new biomarkers or auxiliary diagnostic procedures that can be employed in clinical practice is urgently needed.

Cell adhesion molecules (CAMs) (9–11) are membrane-surface glycoproteins that promote cell-to-cell adhesion as well as cell-to-extracellular matrix adhesion. They are members of the immunoglobulin superfamily (IGSF) and have a variety of physiological functions. Studies have implicated adhesive proteins in multiple pathological linkages of AD, including amyloid plaque degradation, diffusion, and inflammation (12–14). The vascular cell adhesion molecule 1 (VCAM-1) (15) is found on the surface of many different types of cells, including active endothelial cells and macrophages (16). White blood cells contain a ligand, which stimulates them to traverse the vascular endothelium and aggregate toward inflammation. Previous research confirmed that the new role of the hippocampus VCAM-1 in brain aging regulation was directly related to cognitive processes (16). It has also been shown that VCAM-1 in plasma of AD patients is higher than MCI, and this relationship is related to multiple cerebrospinal fluid components (17). Nerve cell adhesion molecule (NCAM) (10) is a glycoprotein found on the cell membrane that regulates axon/dendrite growth, branching, synaptic extension, shape, and cognitive processes (18). It could be the neurobiological basis of AD learning and memory dysfunction. As one of the adhesion structures, activated leukocyte cell adhesion molecule (ALCAM) (19), positioned at the cell junction in the epithelium, can maintain tissue structure stability. Intercellular adhesion molecule 1 (ICAM-1) (11), an inflammatory factor secreted by vascular endothelium, can mediate platelet, white blood cell, and vascular endothelial adhesion. Although it is expressed at low levels in normal cells and tissue, it is rapidly up-regulated once the inflammatory reaction begins. As a result, it plays a critical role in the inflammatory response process.

Whilst all the above-mentioned adhesion molecules are potentially implicated in AD pathogenesis, their diagnostic value as auxiliary markers remain elusive. The present work purposed to evaluate the association between these adhesion molecules and the severity of AD and the level of inflammation. Moreover, their predictive value in clinical practice is assessed. A clinical application of CAMs as auxiliary diagnostic markers is also evaluated to establish whether their combination could improve predictive accuracy.

## Methods

### Study participants

This study enrolled 71 patients with Alzheimer’s disease (AD), 44 patients with mild cognitive impairment (MCI) (20), 18 patients with other dementia (OD) and 28 healthy people (Normal control, NC) screened and diagnosed in the First Affiliated Hospital of Shantou University Medical College between January 2021 and November 2022. All experiments were performed with the informed and overt consent of each participant in accordance with the Clinical Ethics Committee of The First Affiliated Hospital of Shantou University Medical College (2020-115-XZ2, B-2022-232). Clinical screening, cognitive function evaluation, and imaging examination were used to diagnose AD according to the revised version of the NINCDS-ADRDA criteria proposed by the International Working Group (IWG) in 2007 (21). Exclusion criteria included a clinical stroke history three months before admission, an MRI confirmed regional infarction, autoimmune or systemic inflammatory diseases, malignant tumors, and treatment with NSAIDs or steroids as these may influence circulating CAM levels (15, 22).

### Demographic data

All participants were recruited from Shantou, Guangdong Province, China, accompanied by their families, who consented to provide relevant information, including age, gender, and education level, while also accepting the corresponding inspection.

### Cognitive test

In addition to routine outpatient consultations, we administered cognitive tests to all participants using the locally adapted 30-point Mini-Mental State Examination (MMSE) and Montreal Cognitive Assessment (MoCA) (23, 24). Participants were tested in the language they were most comfortable with. The test sequence started with the MMSE, followed by each participant in the MoCA. The MMSE had five subsections (Orientation, Reg indication, Recall, Attention, Calculation, Language, and Praxis). MoCA was divided into seven subsections (Visuospatial/Executive, Naming, Memory and Delayed recall, Attention, Language, Abstraction, and Orientation). The equivalent chapters on “direction” and “sequence subtraction from the beginning” of the two tests were only tested once to avoid familiarity responding to the task in MMSE. Participants were permitted to use personal visual aids (such as glasses). The test was conducted in a room with sufficient light and sound insulation room to facilitate satisfactory face-to-face dialogue.

### Apolipoprotein E genotype analysis

The patients fasted for 8 hours overnight, and blood was collected from their anterior elbow vein into a vacuum tube containing EDTA for DNA extraction. Genotyping was then performed using snapshot SNP typing. rs429358 and rs7412 at exon 4 of the ApoE gene were sequenced to determine ApoE alleles and genotypes. Furthermore, ApoE status was defined as having one or more copies of ϵ2, ϵ3 and ϵ4, whereas ApoE ϵ4 positive status (ApoE4) was validated as having ϵ4/ϵ4; ϵ3/ϵ4, or ϵ2/ϵ4 (25–28). In the experimental steps, 2 ml anticoagulant peripheral venous blood was collected to extract leukocytes. Genomic DNA was extracted from blood samples using the silicon matrix adsorption column method for PCR amplification of target genes, and PCR products were purified. Sequencing and subsequent analysis were performed following the snapshot extension reaction. The following primer sequences were used for genotyping: rs429358-F: AATCGGAACTGGAGGAACAAC; rs429358-R: GATGGCGCTGAGGCCGCGCTC; rs7412-F: AATCGGAACTGGAGGAACAAC; rs7412-R: GATGGCGCTGAGGCCGCGCTC.

### Clinical chemistry analysis

Cell adhesion molecules (CAMs: ALCAM, VCAM-1, NCAM, ICAM-1) and inflammatory factor (IFN-gamma, IL-18, IL-1beta, IL-13, IL-8, IL-7, CCL11, MCP-1, TSLP, IL-10, BDNF, IL-17, IL-5, TREM-1) in plasma of the participants was detected using the multiple liquid chip technology. The plasma levels of Abeta1-42 and Abeta1-40 were detected by liquid-phase flow cytometry (FCM). Differences between CAMs were determined by comparing the natural logarithm (LN) of their concentrations.

### MRI and visual rating scales

A 1.5T MRI scanner was used to scan T1 weighted hippocampal sequence. The Medial Temporal Atrophy (MTA) scale is based on T1 weighted images (29). MTA has been proven in prospective studies in non-demented subjects to predict future dementia, particularly in general and Alzheimer’s disease (30, 31). The MTA scale ranks the degree of atrophy in the hippocampus, parahippocampal gyrus, entorhinal cortex, and the surrounding cerebrospinal fluid spaces from 0 to 4.

### Data analysis

All statistical analyses were performed with the R statistical software (Version R4.2.1) and the R studio interface (Version 2022.07.0).

The relationship between the four CAMs was compared in all participants using the Kruskal-Wallis test (a nonparametric method for testing whether two or more samples originate from the same probability distribution). Furthermore, the correlation of CAM with clinical index (Demographics data, Cognitive test, and MTA) was examined in all subjects. The potential link between ALCAM, VCAM-1, and AD was evaluated based on the correlations between ALCAM, VCAM-1 and Abeta1-42, Abeta1-40, and inflammatory factors.

To give full play to the value of ALCAM and VCAM-1 in AD diagnosis, all participants were divided into a training set and test set at 7:3 (114:47) at random. ALCAM, VCAM-1, Demographics (Education, Age, Gender), Plasma Biomarker (ApoE4), and Cognitive Test (MMSE, MoCA) were all merged to develop a two-way stepwise logistic regression model. AIC (Akaike information criterion, a standard to measure the goodness of statistical model fitting) was gradually reduced until the best model was obtained. The bootstrap-resampling method was used for internal verification in the training set. The resampling number was set to 1000, to generate the Nomograms. The same method was used for external verification of the test set, and the calibration curve of the best model was generated.

## Results

### Increase in plasma ALCAM and VCAM-1 levels is positively linked to the severity of the cognitive decline

This study enrolled 131 patients with cognitive dysfunction, including 71 patients with AD, 44 patients with MCI, and 18 patients with other dementias (OD). In addition, 28 healthy people (Normal control, NC) were enlisted. All participants were clinically diagnosed after the outpatient clinical screening, cognitive function assessment, and imaging. **Table 1** shows a significant difference in age, education, MMSE, MoCA, and the ApoE4 carrier ratio between AD and non-AD (MCI, OD, and NC). Alzheimer’s disease patients were the oldest (72.0 ± 9.01 years) and the least educated (6.75 ± 4.47 years). They also had the lowest MMSE (13.5 ± 8.46) and MoCA (9.73 ± 7.0) scores, but with ApoE4 carrier ratio (47.9%).

**Table 1:** Basic characteristics of study participants: This study recruited 71 patients with Alzheimer’s disease (AD), 44 patients with mild cognitive impairment (MCI), 18 patients with other dementia (OD), and 28 healthy people (Normal control, NC).

Characteristics	AD(n=71)	nonAD				p-value
		MCI (n=44)	OD (n=18)	NC (n=28)	nonAD (n=90)	
Age (years)						
** Mean (SD)**	**72.0 (9.01)**	**66.6 (8.93)**	**70.1 (9.60)**	**60.7 (11.0)**	**72.0 (9.01)**	**<0.001**
** Median [Min,Max]**	**73.0 [48.0, 87.0]**	**66.0 [51.0, 85.0]**	**69.0 [52.0, 85.0]**	**58.0 [48.0, 85.0]**	**73.0 [48.0, 87.0]**	
Gender						
** Male**	**21 (29.6%)**	**16 (36.4%)**	**2 (11.1%)**	**7 (25.0%)**	**25 (27.8%)**	**0.94**
** Female**	**50 (70.4%)**	**28 (63.6%)**	**16 (88.9%)**	**21 (75.0%)**	**65 (72.2%)**	
Education (years)						
** Mean (SD)**	**6.75 (4.47)**	**9.64 (3.36)**	**8.28 (5.09)**	**11.4 (3.92)**	**6.75 (4.47)**	**<0.001**
** Median [Min,Max]**	**6.00 [0, 16.0]**	**9.00 [0, 16.0]**	**9.00 [0, 16.0]**	**12.0 [6.00, 21.0]**	**6.00 [0, 16.0]**	
MMSE						
** Mean (SD)**	**13.5 (8.46)**	**24.7 (6.28)**	**15.2 (7.65)**	**26.9 (6.36)**	**13.5 (8.46)**	**<0.001**
** Median [Min, Max]**	**13.0 [0, 29.0]**	**27.0 [4.00, 30.0]**	**14.0 [1.00, 29.0]**	**28.5 [2.00, 30.0]**	**13.0 [0, 29.0]**	
MOCA						
** Mean (SD)**	**9.73 (7.00)**	**21.0 (2.94)**	**12.3 (6.30)**	**24.0 (4.98)**	**9.73 (7.00)**	**<0.001**
** Median [Min, Max]**	**8.00 [0, 24.0]**	**21.0 [8.00, 26.0]**	**11.5 [1.00, 23.0]**	**25.0 [3.00, 30.0]**	**8.00 [0, 24.0]**	
ApoE4						
** ApoE4 carrier**	**35 (49.3%)**	**10 (22.7%)**	**6 (33.3%)**	**3 (10.7%)**	**35 (49.3%)**	**<0.001**
** ApoE4 non-carrier**	**36 (50.7%)**	**34 (77.3%)**	**12 (66.7%)**	**25 (89.3%)**	**36 (50.7%)**	

The patients with AD were the oldest (72.0 ± 9.01 years) and least educated (6.75 ± 4.47 years). They also had the lowest score in MMSE (13.5 ± 8.46) and MoCA (9.73 ± 7.0) but with the highest ApoE4 carrier ratio (47.9%).

**Figure 1:**
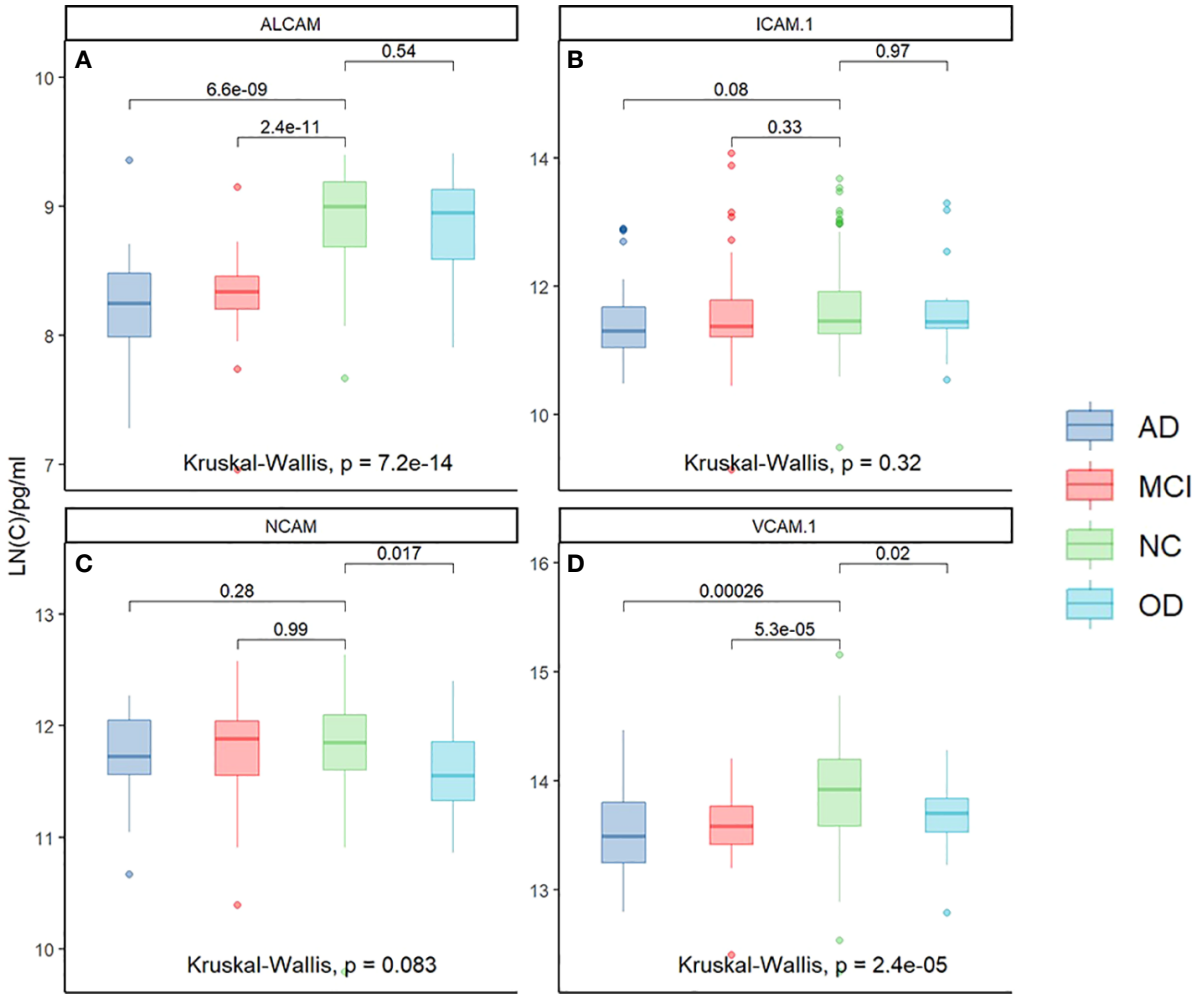
The relationship of Plasma CAMs in different groups: ALCAM (Kruskal-Wallis test, p<0.001) and VCAM-1 (Kruskal-Wallis test, p<0.001) demonstrated significant differences among the four groups. Plasma ALCAM was different between NC and AD (p<0.001), and between MCI and AD (p<0.001). **(A)** There were no significant differences in ALCAM between AD and OD. **(B)** There was no significant difference in the plasma ICAM-1 between the two groups. **(C)** Patients with AD had a higher level of NCAM than OD (p<0.05) but not MCI or NC. **(D)** A higher level of VCAM-1 was reported in the plasma of AD patients than in MCI (p<0.001), NC (p<0.001), and OD (p<0.05). *Y-axis represents the natural logarithm (LN) of CAM concentration.

To understand the changes in plasma CAMs, we detected some of their adhesion molecules, including ALCAM, ICAM-1, NCAM, and VCAM-1 (**Supplementary Table 1**). There was a substantial variation in CAM expression between the four groups, which has been linked to AD. First, the relationship between the four CAMs was compared in all populations using the Kruskal-Wallis test; the analysis revealed that ALCAM (p<0.001) and VCAM-1 (p<0.001) exhibit significant differences across the four groups. Next, we compared the differences between the two groups. As illustrated in **Figure 1**, plasma ALCAM levels differed between NC and AD (p<0.001) and MCI and AD (p<0.001). However, no significant differences were recorded in ALCAM between AD and OD (**Figure 1A**), as well as in the plasma ICAM-1 between the two groups (**Figure 1B**). The findings also revealed that AD patients had higher levels of NCAM than OD patients (p<0.05), but not MCI or NC (**Figure 1C**). Plasma VCAM-1 level of AD patients was higher than in MCI (p<0.001), NC (p<0.001), and OD patients (p<0.05) (**Figure 1D**). Furthermore, VCAM-1 and ALCAM levels in AD peripheral blood were found to be higher than in other groups. VCAM-1 and ALCAM levels were higher in the peripheral blood of AD patients than in other groups, making it beneficial for the differential diagnosis of AD.

**Figure 2:**
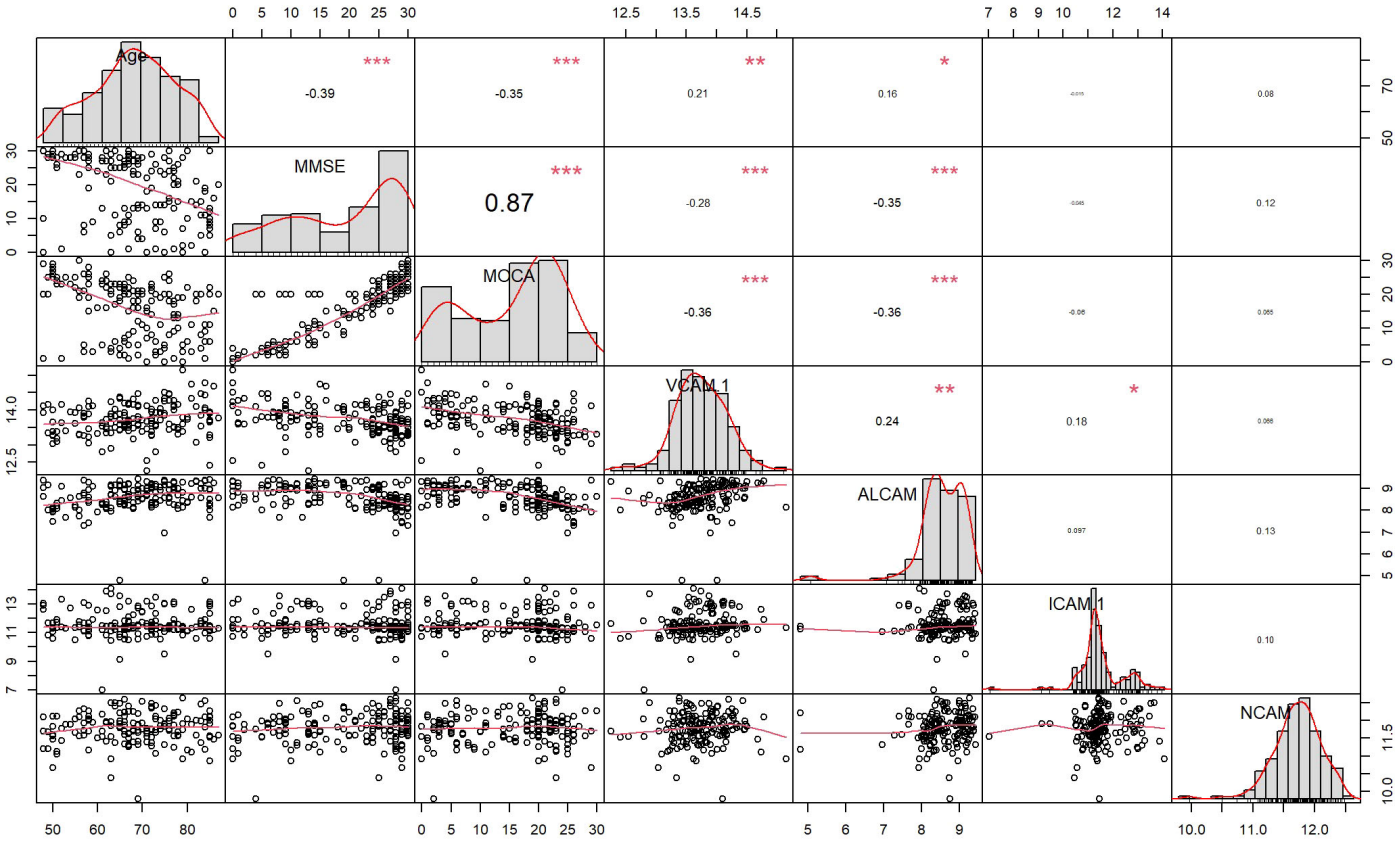
The correlation between four CAMs and Demographics data, Cognitive test (MMSE, MOCA) in all participants: Spearmen correlation analysis revealed a correlation of ALCAM with Age (r=0.16, p=0.0018<0.05), MMSE (r=-0.35, p<0.001), MoCA (r=-0.36, p<0.001), whereas VCAM-1 was correlated with Age (r=0.21, p=0.0085<0.05), MMSE (r=-0.28, p<0.001), MoCA (r=-0.36, p <0.001). * The curve at the lower left corner represents the fitting curve of two factors, the value at the upper right corner represents the correlation coefficient (r -value) of two factors, * represents the significance of correlation (“***”: p-value<0.001, “**”: p-value<0.01, “*”: p-value<0.05, “ “: p-value>0.05), and the diagonal represents the histogram of single factor and corresponding curve.

**Figure 3:**
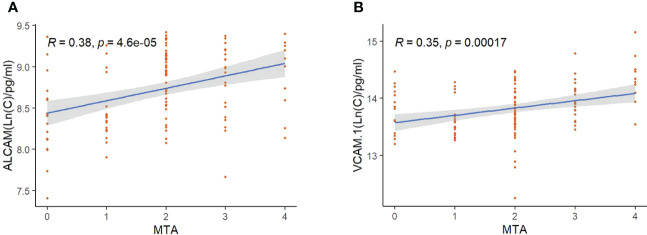
The correlation between ALCAM, VCAM-1, and MTA in all participants: **(A)** Spearmen correlation analysis revealed a significant correlation of ALCAM with MTA (r=0.38, p<0.001). **(B)** Spearmen correlation analysis revealed a significant correlation of VCAM-1 with MTA (r=0.35, p<0.001). * The blue line represents the fitting line. Shadows represent a 95% confidence interval.

To determine the clinical correlation, we compared plasma CAMs with MMSE, MoCA, and age in all subjects. VCAM-1 and ALCAM were negatively correlated with cognitive functions and age (**Figure 2**). Higher levels of VCAM-1 and ALCAM were associated with a lower MMSE score (VCAM-1: r=-0.28, p<0.001, ALCAM: r=-0.35, p<0.001), lower MoCA score (VCAM-1: r=-0.36, p <0.001, ALCAM: r=-0.36, p<0.001) and an older age (VCAM-1: r=0.21, p=0.0085, ALCAM: r=0.16, p=0.0018). In addition, we investigated their (VCAM-1 and ALCAM) correlation with MTA in all groups, which is a critical index for assessing the degree of atrophy in the medial temporal lobe (**Supplementary Table 1**). The analysis revealed that they were significantly negatively correlated with MTA score (**Figure 3**: VCAM-1: r= 0.38, p<0.001, ALCAM: r= 0.35, p<0.001). The findings suggest that both adhesion molecules could be used to predict disease severity. The higher the plasma concentration of ALCAM and VCAM-1, the higher the atrophy of the medial temporal lobe structure. However, neither ICAM-1 nor NCAM could correlate significantly with the clinical index (MMSE, MoCA, MTA, Age).

### Plasma levels of VCAM-1 and ALCAM in plasma are correlated with inflammatory factors

**Figure 4:**
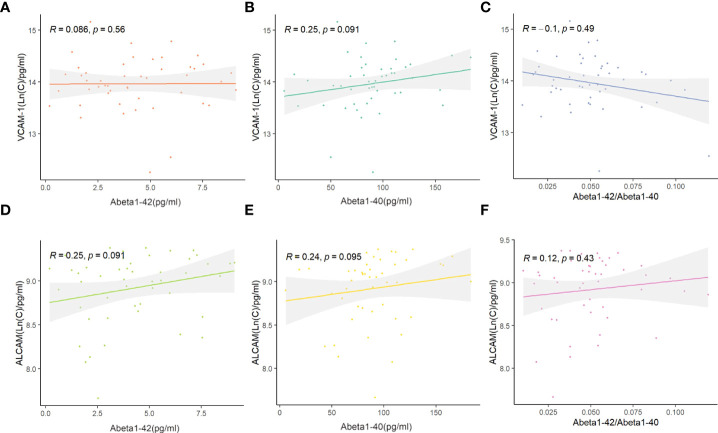
The correlation between ALCAM, VCAM-1, and Abeta1-40, Abeta1-42, Abeta1-40/Abeta1-42 in participants with AD: **(A–C)** Spearmen correlation analysis demonstrated that VCAM-1 was not significantly correlated with Abeta1-42 (p-value > 0.05, **A**), Abeta1-40 (p-value > 0.05, **B**) and Abeta1-40/Abeta1-42 (p-value > 0.05, **C**). **(D–F)** Spearmen correlation analysis demonstrated that ALCAM was not significantly correlated with Abeta1-42 (p-value > 0.05, **D**), Abeta1-40 (p-value > 0.05, **E**) and Abeta1-40/Abeta1-42 (p-value > 0.05, **F**).

Abeta1-40 and Abeta1-42 detection in the plasma of AD was performed to reveal the link between VCAM-1 or ALCAM and pathological changes and investigate their potential predictive mechanisms in AD (**Supplementary Table 2**). Plasma VCAM-1 and ALCAM had no significant correlation with plasma Abeta1-40, Abeta1-42, or ratio of Abeta1-42 to Abeta1-40 (Abeta1-42/Abeta1-40) (**Figures 4A–F**).

**Figure 5:**
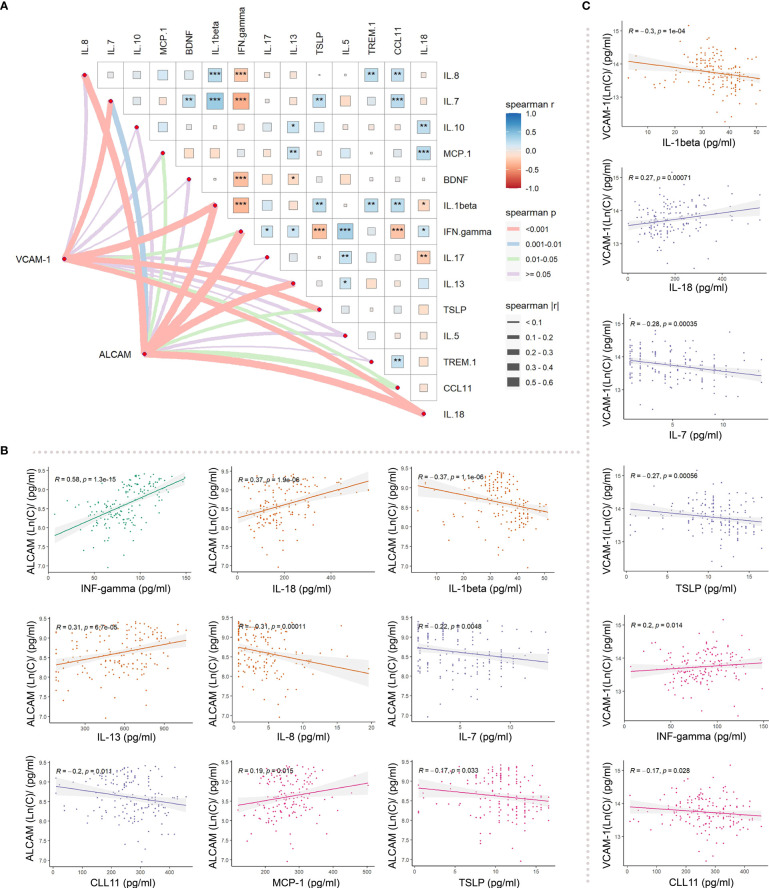
Differences in the correlation between ALCAM, VCAM_1, and inflammatory factors in participants with AD: **(A)** Spearman correlation analysis revealed an association of ALCAM with IFN-gamma, IL-18, IL-1beta, IL-13, IL-8, IL-7, CCL11, MCP-1 and TSLP. VCAM-1 was correlated with IL-1beta, IL-18, IL-7, TSLP, IFN. gamma, and CCL11. **(B)** ALCAM was significantly correlated with IFN- gamma (r=0.58, p<0.001), IL-18 (r=0.37, p<0.001), IL-1beta (r=-0.37, p<0.001), IL-13 (r=0.31, p<0.001), IL-8 (r=-0.31, p<0.001), and IL-7 (r=-0.22, p<0.001). **(C)** VCAM-1 was significantly correlated with IL-1beta (r=-0.3, p<0.001), IL-18 (r=0.27, p<0.001), IL-7 (r=-0.28, p<0.001), and TSLP (r=-0.27, p<0.001). * represents the significance of correlation (“***”: p-value<0.001, “**”: p-value<0.01, “*”: p-value<0.05, “ ”: p-value>0.05).

Moreover, we detected several inflammatory factors in the plasma of AD, including IFN-gamma, IL-18, IL-1beta, IL-13, IL-8, IL-7, CCL11, MCP-1, TSLP, IL-10, BDNF, IL-17, IL-5, and TREM-1 (**Supplementary Table 2**). VCAM-1 and ALCAM were significantly correlated with several inflammatory factors in plasma (**Figure 5A**). Through Spearman correlation analysis, ALCAM was shown to be significantly correlated with IFN-gamma (r=0.58, p<0.001), IL-18 (r=0.37, p<0.001), IL-1beta (r=-0.37, p<0.001), IL-13 (r=0.31, p<0.001), IL-8 (r=-0.31, p<0.001), and IL-7 (r=-0.31, p<0.001) (**Figure 5B**). Meanwhile, VCAM-1 was significantly correlated with IL-1beta (r=-0.3, p<0.001), IL-18 (r=0.27, p<0.001), IL-7 (r=-0.28, p<0.001), TSLP (r=-0.27, p<0.001) (**Figure 5C**). These data indicate that CAMs may reflect a pathological inflammatory state, which could serve as a critical index for predicting AD severity.

### Clinical Predictive Value of VCAM-1 and ALCAM

**Figure 6:**
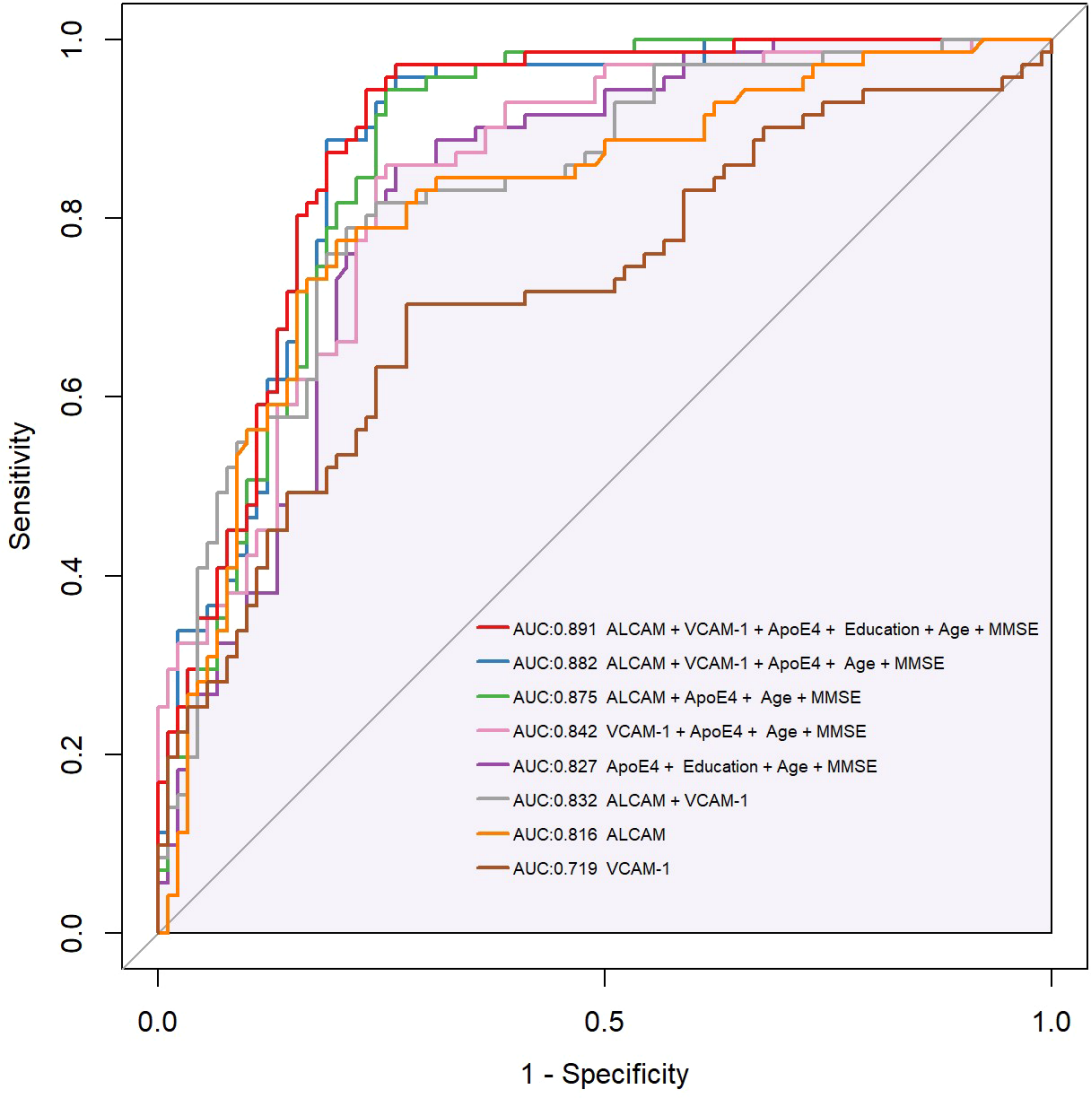
Compound ROC curve of all types of models. CAMs (ALCAM, VCAM-1) were combined with demographics (Education, Age), plasma biomarker (ApoE4), and cognitive tests (MMSE, MoCA) to establish a new diagnostic model. AIC (Akaike information criterion) was gradually reduced through two-way stepwise logistic regression. The best model, integrating ApoE4, Age, Education, MMSE, VCAM-1, and ALCAM (AUC:0.891, AIC 146.9), and a compound ROC curve were eventually generated. Purple shadow represents the basic model, integrating ApoE4, age, education, and MMSE (AUC:0.827, AIC 173). * Y-axis of the ROC curve represents the value of sensitivity, and X-axis represents the value of 1 minus Specificity (1-Specificity).

Previous findings revealed that VCAM-1 and ALCAM were useful in the differential diagnosis of AD. In this view, we employed VCAM-1 and ALCAM as independent predictors. To predict AD, multiparameter regression analysis was performed with demographics (age, gender), cognitive test (MMSE, MoCA), ApoE4 genotype, ALCAM, and VCAM-1. The most predictive single variable or multiple variable combinations for AD diagnosis were also compared. AIC was gradually reduced by two-way stepwise regression (**Table 2**). Eventually, the best model was developed, based on the combination of ApoE4, age, education, MMSE, VCAM-1, and ALCAM (AUC: 0.891, AIC: 146.9, Model 1) (**Figure 6**). ALCAM and VCAM-1 contributed the most to the best prediction model. ALCAM and VCAM-1 solely had an AUC of 0.832 (Model 6) in AD prediction, which was higher than the results of the basic model (age, education, MMSE, and ApoE4) (AUC: 0.827, AIC: 173, Model 5). ALCAM and VCAM-1 combination with traditional information yielded a significantly higher predictive value (**Supplementary Figure 1A**, p=0.012<0.05). The best model was more accurate than the basic model (**Supplementary Figure 1B**, p=0.006<0.05). The AUC of the best model was nearly similar to the combined MTA scale of the basic model (**Supplementary Figure 1C**, p=0.128>0.05). These findings suggested that the model combined with VCAM-1 and ALCAM (Model 1, the best Model)could accurately predict AD even without MRI data.

**Table 2:** Parameters of each model under two-way stepwise logistic regression: Area under curve (AUC), Akaike information criterion (AIC), and the P value of statistical difference compared with Model 5 (p-VALUE).

Model Number	Model Content	AUC(95%CI)	AIC	p-VALUE
**Model 1(bes)**	**ALCAM + VCAM-1 + ApoE4 + Education + Age + MMSE**	**0.891(0.841-0.941)**	**146.9**	0.006 **
**Model 2**	**ALCAM + VCAM-1 + ApoE4 + Age + MMSE**	**0.882(0.829-0.934)**	**147.3**	0.027 *
**Model 3**	**ALCAM + ApoE4 + Age + MMSE**	**0.875(0.821-0.929)**	**149.4**	0.043 *
**Model 4**	**VCAM-1 + ApoE4 + Age + MMSE**	**0.842(0.782-0.902)**	**166.8**	**0.388**
**Model 5(basic)**	**ApoE4 + Education + Age + MMSE**	**0.827(0.764-0.891)**	**173**	**-**
**Model 6**	**ALCAM + VCAM-1**	**0.832(0.769-0.895)**	**166.6**	**0.913**
**Model 7**	**ALCAM**	**0.816(0.748-0.884)**	**170.8**	**0.789**
**Model 8**	**VCAM-1**	**0.719(0.638-0.801)**	**203.8**	0.027 *

*The “*” represents a significant correlation (“***”: p-value<0.001, “**”: p-value<0.01, “*”: p-value<0.05, “ ”: p-value>0.05).

**Figure 7:**
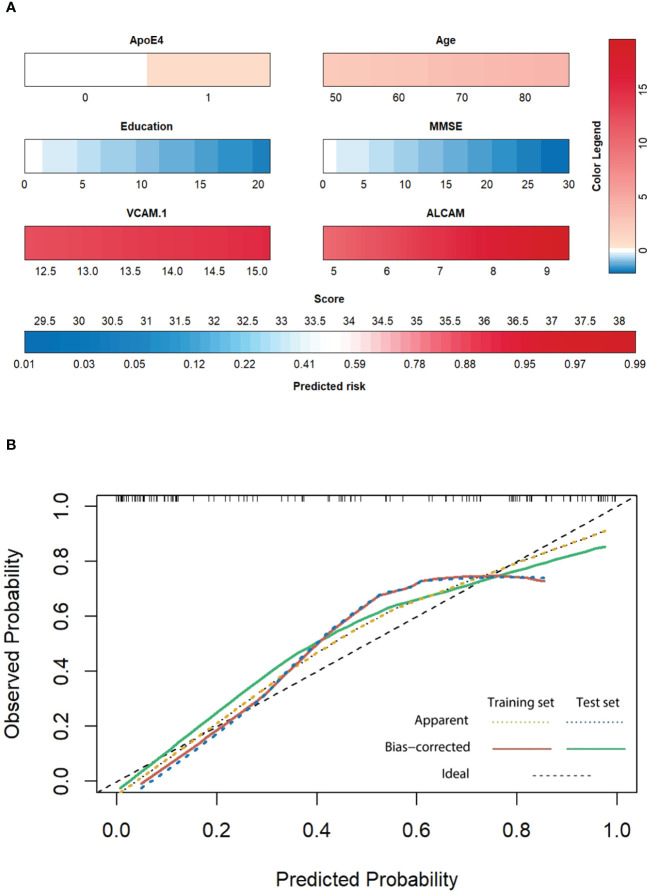
**(A)** The Nomogram of the best model. Each indicator is the collected measured value (below the indicator frame), which is converted into the corresponding score according to the color and color legend (upper right). The total score is obtained by adding all factors, and the corresponding forecast risk below is converted into the specific forecast probability. **(B)** The calibration curve of the training set and test set: Patients were divided into the training set and test set according to 7:3 (114:47), followed by internal verification with the bootstrap resampling method within the training set. The number of resampling was set to 1000. External verification was performed with the same method for the testing set, and a calibration curve of the best model was generated. The X-axis represents the prediction probability, the Y-axis represents the observation probability, and the black dotted line represents the ideal curve. The yellow dotted line represents the actual observation curve of the training set, the red solid line represents the calibration curve of the training set (n=114, Mean absolute error=0.051, Mean squared error=0.0041, 0.9 Quantity of absolute error=0.105), the blue dotted line represents the actual observation curve of the test set, and the green solid line represents the calibration curve of the test set (n=47, Mean absolute error=0.061, Mean squared error=0.00576, 0.9 Quantity of absolute error=0.124).

Finally, we used the parameters in the best model (ApoE4, age, education, MMSE, VCAM-1, and ALCAM) to construct a personalized risk prediction model for AD. The enrolled subjects were divided into training and test sets in a 7:3 ratio (114/47); internal verification was performed with a bootstrap-resampling approach within the training set. Resampling was set to 1000 to generate Nomograms (**Figure 7A**). A similar method was employed for external verification of the test set, and a calibration curve of the best model was generated. Intriguingly, the top prediction model had the best predictive probability (**Figure 7B**).

## Discussion

This study looked into four different CAM types. ALCAM and VCAM-1 may aid in differential diagnosis based on a correlation analysis with clinical indicators. The composite prediction model of ALCAM, VCAM-1, age, education, ApoE4, and MMSE was demonstrated to have a higher prediction accuracy for AD. This prediction model is of great promise as it can achieve the same prediction accuracy in the absence of imaging diagnosis. In view of these findings, ALCAM and VCAM-1 met the requirement for biological markers in large-scale screening tests.

Inflammation is heavily linked to the onset and progression of AD, an age-related neurodegenerative disease (14, 32). Inflammation is evident in brain tissue, cerebrospinal fluid, and peripheral blood. Previous evidence indicates that traditional biomarkers are largely associated with amyloid plaques or Tau protein phosphorylation, and inflammation in AD is rarely used as a diagnostic tool (20, 28). The model based on cell adhesion molecules exhibited a comparable prediction value to the classic MRI-based AD model. These findings strongly demonstrated that cell adhesion molecules could be employed in the future for clinical differential diagnosis of AD.

ALCAM is predominantly expressed in leukocytes and thymic epithelial cells. It binds to the lymphocyte antigen CD6 and interacts with inflammatory factors to mediate immune cell adhesion and regulate T-cell development and function (33). ALCAM has previously been utilized to evaluate tumor invasiveness and immunity; however, it has received little attention in AD research (34). Until recently, only one study has linked the expression of ALCAM-coding genes to AD. Our research group was the first to note a link between plasma ALCAM expression and AD. While our evidence was circumstantial and did not establish causation, this finding reflected the role of peripheral inflammatory factors in AD progression. Moreover, the VCAM-1 protein has also been extensively investigated in AD and it is thought to play a role in T-cell invasion and inflammatory responses (35–37). In the present work, we found a strong correlation of VCAM-1 protein with cognitive functions and the degree of medial temporal lobe atrophy. VCAM-1 had a higher predictive value in AD from this standpoint; however, our data demonstrated that ALCAM had a stronger contribution to AD prediction than VCAM-1. The traditional prediction model had a higher predictive value after merging the parameters of VCAM-1 and ALCAM. As a result, different CAMs indicate different inflammatory pathological processes, and combining them can provide a more comprehensive view of ‘the inflammatory state of AD. Plasma inflammatory factors are expected to vary substantially in response to other clinical stimuli, and their specificity remains unknown. Additionally, because they are generally low in concentration, sensitive detection methods such as mass spectrometry is warranted. As a result, utilizing inflammatory factors to predict AD may be not suitable. Plasma CAM could be used to predict AD.

The limitation of this experiment was that the predictive value of adhesion molecules was evaluated by clinical diagnosis; our clinical diagnosis model was based on clinical symptoms, neuropsychological assessment, and MRI (atrophy degree of medial temporal lobe structure). However, our subsequent study will test the accuracy of the CAM prediction model using a biomarker model (Clinical assessment, MRI, and biomarkers).

Cell adhesion molecules could be seen from our perspective as a screening indicator but not a diagnostic indicator. In other words, screening for patients with cognitive impairments was implemented to improve screening efficiency rather than to make a definitive diagnosis.

Our research preliminarily assessed the predictive accuracy and demonstrated its potential as a biological marker. Future research is anticipated to develop a basic tool for the differential diagnosis of AD.

## Data availability statement

The original contributions presented in the study are included in the article/**Supplementary Material**. Further inquiries can be directed to the corresponding author.

## Ethics statement

The studies involving human participants were reviewed and approved by The Clinical Ethics Committee of The First Affiliated Hospital of Shantou University Medical College (2020-115-XZ2, B-2022-232). The patients/participants provided their written informed consent to participate in this study. Written informed consent was obtained from the individual(s) for the publication of any potentially identifiable images or data included in this article.

## Author contributions

JC and A-XD: data collection, manuscript writing, and design of this article. N-LW: supervision, funding, and manuscript editing. C-HL: ApoE genotyping procedure. TH, H-XL, Z-JL, NK, X-YP, K-XL, Z-DZ: clinical data analysis. S-LX, XZ, HP, X-FY: clinical evaluation. All authors contributed to the article and approved the submitted version.

## Supplementary material

Supplementary Table 1 CAMs and MTA in four groups.

Supplementary Table 2 Plasma factors in patients with AD.

Supplementary Figure 1 The correlation between ALCAM, VCAM_1 and inflammatory factors: **(A)** ROC curve between Model 6 (ALCAM +VCAM-1) and Best Model has a statistical difference (p<0.05). **(B)** ROC curve between Best Model and Basic Model has a significant difference (p<0.01). **(C)** ROC curve between the best model and the model combined with MTA (ApoE4 + Age + Education + MMSE + MTA) (AUC: 0.912) has no significant difference (p>0.05). * Y-axis of the ROC curve represents the value of sensitivity, and X-axis represents the value of (1-Specificity).
