# Supplementary material for: Increase of ALCAM and VCAM-1 in the plasma predicts the Alzheimer’s disease
Source: Front Immunol. 2023 Jan 4;13:1097409. doi: 10.3389/fimmu.2022.1097409 (PMC9846483; doi:10.3389/fimmu.2022.1097409)
Supplement: Supplementary Table 1 — CAMs and MTA in four groups. [file Table_1.docx]

|  |  |  |  |  |  |  |
| --- | --- | --- | --- | --- | --- | --- |
| **Characteristics** | **AD** | **MCI** | **OD** | **NC** | **Total** |  |
|  |  |  |  |  |  |  |
| **ALCAM (LN(C)pg/ml)** |  |  |  |  |  |  |
| **Mean (SD)** | **8.90 (0.383)** | **8.23 (0.619)** | **8.62 (1.04)** | **8.20 (0.451)** | **8.57 (0.648)** |  |
| **Median [Min, Max]** | **9.00[7.66, 9.40]** | **8.33[4.80, 9.15]** | **8.95[4.80, 9.41]** | **8.25[7.28, 9.36]** | **8.59[4.80, 9.41]** |  |
| **ICAM-1 (LN(C)pg/ml)** |  |  |  |  |  |  |
| **Mean (SD)** | **11.7 (0.803)** | **11.6 (0.895)** | **11.6 (0.733)** | **11.3 (1.09)** | **11.6 (0.880)** |  |
| **Median [Min, Max]** | **11.5[9.48, 13.7]** | **11.4[9.13, 14.1]** | **11.5[10.5, 13.3]** | **11.3[7.03, 12.9]** | **11.4[7.03, 14.1]** |  |
| **NCAM (LN(C)pg/ml)** |  |  |  |  |  |  |
| **Mean (SD)** | **11.8 (0.450)** | **11.8 (0.435)** | **11.6 (0.378)** | **11.7 (0.393)** | **11.8 (0.431)** |  |
| **Median [Min, Max]** | **11.9[9.79, 12.6]** | **11.9[10.4, 12.6]** | **11.6[10.9, 12.4]** | **11.7[10.7, 12.3]** | **11.8[9.79, 12.6]** |  |
| **VCAM-1(LN(C)pg/ml)** |  |  |  |  |  |  |
| **Mean (SD)** | **13.9 (0.487)** | **13.6 (0.325)** | **13.7 (0.340)** | **13.6 (0.418)** | **13.7 (0.446)** |  |
| **Median [Min, Max]** | **13.9[12.2, 15.2]** | **13.6[12.4, 14.2]** | **13.7[12.8, 14.3]** | **13.5[12.8, 14.5]** | **13.7[12.2, 15.2]** |  |
| **MTA** |  |  |  |  |  |  |
| **Mean (SD)** | **2.60 (0.773)** | **1.04 (0.774)** | **1.50 (0.674)** | **0 (0)** | **1.88 (1.15)** |  |
| **Median [Min, Max]** | **2.00[1.00, 4.00]** | **1.00[0, 3.00]** | **2.00[0, 2.00]** | **0 [0, 0]** | **2.00 [0, 4.00]** |  |
|  |  |  |  |  |  |  |
