# Supplementary material for: Increase of ALCAM and VCAM-1 in the plasma predicts the Alzheimer’s disease
Source: Front Immunol. 2023 Jan 4;13:1097409. doi: 10.3389/fimmu.2022.1097409 (PMC9846483; doi:10.3389/fimmu.2022.1097409)
Supplement: Supplementary Table 2 — Plasma factors in patients with AD. [file Table_2.docx]

|  |  |  |
| --- | --- | --- |
| **plasma factors (pg/ml)** | **Mean (SD)** | **Median [Min, Max]** |
| **IFN-gamma** | **97.8 (23.0)** | **98.5 [29.6, 149]** |
| **IL-18** | **266 (203)** | **230 [30.4, 1040]** |
| **IL-1beta** | **31.9 (6.50)** | **32.6 [5.75, 43.8]** |
| **IL-13** | **596 (232)** | **617 [78.7, 1070]** |
| **IL-8** | **4.61 (8.35)** | **2.98 [0.652, 52.9]** |
| **IL-7** | **3.98 (3.28)** | **3.27 [0.782, 13.5]** |
| **CCL11** | **242 (92.1)** | **244 [9.88, 418]** |
| **MCP-1** | **396 (835)** | **267 [150, 7080]** |
| **TSLP** | **9.99 (3.41)** | **10.6 [0.473, 16.6]** |
| **IL.10** | **12.1 (4.06)** | **13.0 [0.706, 18.8]** |
| **BDNF** | **3770 (2510)** | **3020 [407, 10900]** |
| **IL.17** | **10.5 (6.39)** | **10.0 [2.07, 26.1]** |
| **IL.5** | **9.70 (3.69)** | **10.5 [1.06, 15.7]** |
| **TREM.1** | **211 (73.8)** | **225 [16.8, 424]** |
| **Abeta1-40** | **88.6 (36.4)** | **87.8 [5.90, 184]** |
| **Abeta1-42** | **4.29 (2.32)** | **4.01 [0.193, 9.09]** |
| **Abeta1-42/1-40** | **0.0503 (0.0233)** | **0.0492 [0.0102, 0.119]** |
|  |  |  |
