# Supplementary figures and images for: Increase of ALCAM and VCAM-1 in the plasma predicts the Alzheimer’s disease
Source: Front Immunol. 2023 Jan 4;13:1097409. doi: 10.3389/fimmu.2022.1097409 (PMC9846483; doi:10.3389/fimmu.2022.1097409)

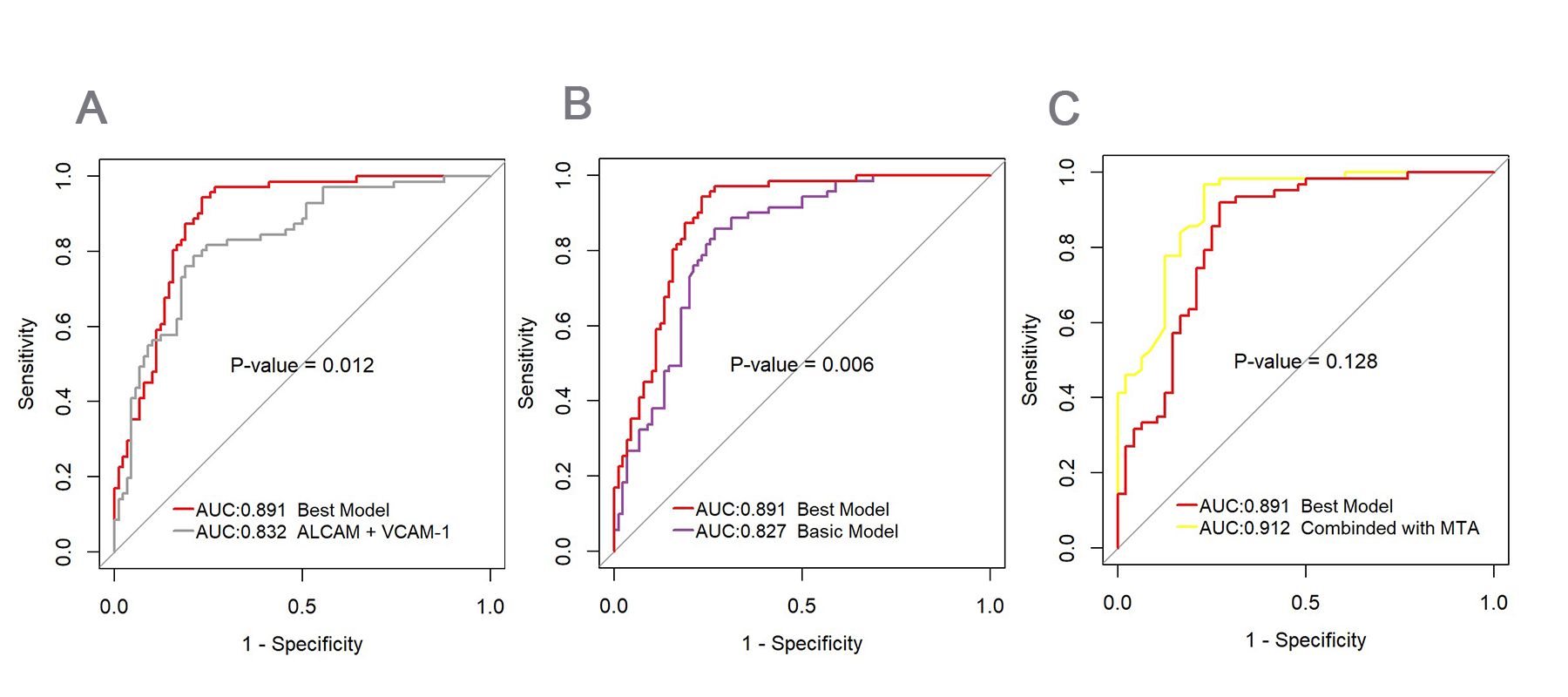

Supplement: Supplementary Figure 1 — The correlation between ALCAM, VCAM_1 and inflammatory factors: (A) ROC curve between Model 6 (ALCAM +VCAM-1) and Best Model has a statistical difference (p<0.05). (B) ROC curve between Best Model and Basic Model has a significant difference (p<0.01). (C) ROC curve between the best model and the model combined with MTA (ApoE4 + Age + Education + MMSE + MTA) (AUC: 0.912) has no significant difference (p>0.05). * Y-axis of the ROC curve represents the value of sensitivity, and X-axis represents the value of (1-Specificity). [file Image_1.tif]
